# Low α2-Plasmin Inhibitor Antigen Levels on Admission Are Associated With More Severe Stroke and Unfavorable Outcomes in Acute Ischemic Stroke Patients Treated With Intravenous Thrombolysis

**DOI:** 10.3389/fcvm.2022.901286

**Published:** 2022-07-15

**Authors:** Edina Gabriella Székely, Rita Orbán-Kálmándi, István Szegedi, Éva Katona, Barbara Baráth, Katalin Réka Czuriga-Kovács, Linda Lóczi, Nikolett Vasas, István Fekete, Klára Fekete, Ervin Berényi, László Oláh, László Csiba, Zsuzsa Bagoly

**Affiliations:** ^1^Division of Clinical Laboratory Sciences, Department of Laboratory Medicine, Faculty of Medicine, Kálmán Laki Doctoral School, University of Debrecen, Debrecen, Hungary; ^2^Department of Neurology, Faculty of Medicine, University of Debrecen, Debrecen, Hungary; ^3^Department of Radiology, Faculty of Medicine, University of Debrecen, Debrecen, Hungary; ^4^ELKH Cerebrovascular and Neurodegenerative Research Group, Debrecen, Hungary

**Keywords:** α2-plasmin inhibitor, acute ischemic stroke, fibrinolysis, intracerebral hemorrhage (ICH), outcome, recombinant tissue plasminogen activator (rt-PA), thrombolysis

## Abstract

**Background:**

Intravenous administration of recombinant tissue plasminogen activator (rt-PA) fails to succeed in a subset of acute ischemic stroke (AIS) patients, while in approximately 6–8% of cases intracerebral hemorrhage (ICH) occurs as side effect.

**Objective:**

Here, we aimed to investigate α2-plasmin inhibitor (α2-PI) levels during thrombolysis and to find out whether they predict therapy outcomes in AIS patients.

**Patients/Methods:**

In this prospective, observational study, blood samples of 421 AIS patients, all undergoing i.v. thrombolysis by rt-PA within 4.5 h of their symptom onset, were taken before and 24 h after thrombolysis. In a subset of patients (*n* = 131), blood was also obtained immediately post-lysis. α2-PI activity and antigen levels were measured by chromogenic assay and an in-house ELISA detecting all forms of α2-PI. α2-PI Arg6Trp polymorphism was identified in all patients. Stroke severity was determined by NIHSS on admission and day 7. Therapy-associated ICH was classified according to ECASSII. Long-term outcomes were defined at 3 months post-event by the modified Rankin Scale (mRS).

**Results:**

Median α2-PI activity and antigen levels showed a significant drop immediately post-lysis and increased to subnormal levels at 24 h post-event. Admission α2-PI levels showed a significant negative stepwise association with stroke severity. Patients with favorable long-term outcomes (mRS 0–1) had significantly higher admission α2-PI antigen levels (median:61.6 [IQR:55.9–70.5] mg/L) as compared to patients with poor outcomes (mRS 2–5: median:59.7 [IQR:54.5–69.1] and mRS 6: median:56.0 [IQR:48.5–61.0] mg/L, *p* < 0.001). In a Kaplan–Meier survival analysis, patients with an α2-PI antigen in the highest quartile on admission showed significantly better long-term survival as compared to those with α2-PI antigen in the lowest quartile (HR: 4.54; 95%CI:1.92–10.8, *p* < 0.001); however, in a multivariate analysis, a low admission α2-PI antigen did not prove to be an independent risk factor of poor long-term outcomes. In patients with therapy-related ICH (*n* = 34), admission α2-PI antigen levels were significantly, but only marginally, lower as compared to those without hemorrhage.

**Conclusions:**

Low α2-PI antigen levels on admission were associated with more severe strokes and poor long-term outcomes in this cohort. Our results suggest that in case of more severe strokes, α2-PI may be involved in the limited efficacy of rt-PA thrombolysis.

## Introduction

Stroke is a leading cause of death and the major cause of adult disability worldwide, representing a substantial socio-economic burden (1). Eighty percent of all strokes are of ischemic origin, when the patient's only chance of recovery depends on the successful opening of the occluded vessel within a relatively short time frame. As of today, two evidence-based therapies are available for this purpose. Currently, the cornerstone pharmacological therapy for the restoration of blood flow is intravenous recombinant tissue-type plasminogen activator (rt-PA), which must be administered within 4.5 h of symptom onset (2–4). Endovascular therapy known as mechanical thrombectomy is now the standard care for patients in case of large vessel occlusions (LVO) who present within 24 h of stroke symptom onset (5, 6). However, only ~20% of stroke patients have LVO, and delivering treatment to such patients within a rapid time frame is challenging, as the procedure can only be performed in highly specialized centers. Consequently, intravenous thrombolysis using rt-PA remains the mainstay therapy of AIS (2–4). Despite the unquestionable benefit of rt-PA in AIS treatment, this therapy is not a remedy for all. Thrombolysis is only effective in ~30–40% of patients, and its efficacy decreases greatly in case of LVO (7). Besides recanalization failure, about 6–8% of patients develop intracranial hemorrhage (ICH) as side effect, despite taking all precautionary steps to minimize bleeding risk (8). As of today, inefficacy of treatment or therapy-associated ICH cannot be foreseen at the initiation of thrombolysis and their occurrence remains unexplained (9). As the fibrinolytic system provides the very basis of the mechanism of thrombolysis, it can be surmised that individual factors altering lysis susceptibility are likely to be key contributors to recanalization failure and bleeding complications. Surprisingly, our knowledge to date regarding this aspect is limited to a few reports (10–15).

The process of fibrinolysis is tightly regulated on multiple levels by activators, inhibitors, cofactors, and receptors (12, 16, 17). The action of rt-PA during thrombolysis is to generate plasmin from its precursor, plasminogen. The inhibition of rt-PA mainly occurs by the action of plasminogen activator inhibitor 1 (PAI-1). The effect of plasmin itself is regulated by α2-plasmin inhibitor (α2-PI) that binds rapidly and irreversibly to plasmin, forming a stable 1:1 complex, in which the activity of the enzyme is effectively neutralized (18–20). During clot formation, when factor XIII (FXIII) becomes activated, it cross-links α2-PI into the fibrin clot, where it can directly and effectively inhibit plasmin (16, 17, 21, 22). Additional regulatory proteins also exist that influence the ability of plasminogen to bind to fibrin and therefore hinder fibrinolysis (i.e., thrombin activatable fibrinolysis inhibitor: TAFI). We and others in previous clinical studies have looked at the relation of specific elements of fibrinolysis (e.g., PAI-1, plasminogen, TAFI, and FXIII) and AIS thrombolysis outcome, but these studies could not identify the essential factors or mechanisms driving recanalization failure or post-lysis hemorrhage based on admission blood samples (11, 12, 14, 23, 24). Interestingly, although α2-PI is a key regulator of fibrinolysis, a potential association between α2-PI levels and thrombolysis outcomes has not been investigated in a relatively large AIS patient cohort, as yet. Moreover, changes in α2-PI levels during AIS thrombolysis and their effect on outcomes have not been tested in a cohort of more than 100 AIS patients as yet, despite the fact that α2-PI activity is expected to decline rapidly during the administration of rt-PA (25, 26).

In the circulation, α2-PI undergoes both amino-terminal (N-terminal) and carboxy-terminal (C-terminal) proteolytic modifications, leading to a variety of circulating α2-PI molecules with modified activities (20). Approximately 70% of circulating α2-PI is N-terminally cleaved by antiplasmin cleaving enzyme (APCE, also known as soluble fibroblast activation protein: sFAP) (27, 28). The N-terminal cleavage has been shown to affect the extent of α2-PI cross-linking to fibrin by activated FXIII (FXIIIa) to a great extent (17, 21, 29). The N-terminal heterogeneity of α2-PI is affected by a common polymorphism of the SERPINF2 gene (p.Arg6Trp, rs2070863) (30). The C-terminus of α2-PI is also posttranslationally modified, although the enzyme responsible for the cleavage has not been identified as yet. The uncleaved, longer form that can bind plasminogen (PB-α2-PI) constitutes about 65% of circulating α2-PI, while the other form fails to bind plasminogen (NPB-α2-PI) (31). Importantly, it has been shown that cross-linking of α2-PI to fibrin by FXIIIa primarily involves PB-α2-PI and C-terminal cleavage of the circulating protein regulates α2-PI activity (20). It has been known for decades that congenital or acquired α2-PI deficiency leads to a bleeding disorder, while increased levels of α2-PI have been more recently shown to be associated with the increased risk of AIS and venous thrombosis (32). Based on this, one can surmise that α2-PI could play an important regulatory role in the outcome of AIS thrombolysis by rt-PA.

Here, we aimed to test α2-PI activity and antigen levels during the course of intravenous thrombolysis and a potential association between α2-PI levels, stroke severity, and treatment outcomes. We also aimed to test the effect of α2-PI Arg6Trp polymorphism on α2-PI levels and on thrombolysis outcomes. We report that low α2-PI antigen levels on admission were associated with more severe strokes and poor long-term outcomes in the tested cohort of 421 AIS patients. Our results suggest that in case of more severe strokes, α2-PI may be involved in the limited efficacy of rt-PA thrombolysis. α2-PI Arg6Trp polymorphism had no effect on α2-PI activity or antigen levels and showed no association with stroke severity, etiology, or outcomes.

## Materials and Methods

### Patients

In this prospective observational study, adult AIS patients, all within 4.5 h of stroke onset and eligible for intravenous thrombolysis, were enrolled (Department of Neurology, Faculty of Medicine, University of Debrecen, Hungary). Patient enrollment was initiated in March 2011 and was completed in April 2019. Intravenous thrombolytic therapy was applied according to the 2008 European Stroke Organization (ESO) guidelines for rt-PA (Alteplase, Boehringer Ingelheim, Germany) administration (33). The inclusion and exclusion criteria of patients were identical to standard criteria of thrombolysis as described in the ESO 2008 guideline. None of the patients included in the cohort were eligible for mechanical thrombectomy: thrombectomy was either unavailable at the time of enrollment, or the patient was unsuitable to perform the procedure. The presence of AIS was diagnosed based on clinical symptoms and imaging using non-contrast computerized tomography (CT) scan and CT angiography (CTA). CT images taken on admission and 24 h post-lysis were analyzed simultaneously by three independent investigators, and the Alberta Stroke Program Early CT Scores (ASPECTS) were calculated (34). For each patient, the time of symptom onset, demographic and clinical characteristics (age, sex, BMI, previous medications, history of cerebrovascular and cardiovascular diseases, and cerebrovascular risk factors including smoking) were registered on admission. Stroke severity was determined by the National Institutes of Health Stroke Scale (NIHSS) on admission and day 7 after therapy (35). Trial of ORG 10172 in Acute Stroke Treatment (TOAST) criteria was used to identify the etiology of stroke (36). Patients were followed, and long-term functional outcomes were determined at 3 months after the stroke event using the modified Rankin Scale (mRS) (37).

The following outcomes and safety endpoint were registered in this study: 1. Short-term outcome at 7 days post-event: a decrease in NIHSS score by at least 4 points or to 0 was defined as favorable outcome (neurologic improvement), while an increase in NIHSS score by at least 4 points was defined as unfavorable outcome (3, 37). 2. Long-term outcome at 90 days post-event: mRS 0–1 was defined as favorable long-term outcome. 3. Therapy-associated intracerebral hemorrhage (ICH): symptomatic (SICH) or asymptomatic (aSICH) using the European Cooperative Acute Stroke Study (ECASS) II criteria, as observed on CT scans at 24 h post-lysis (38).

### Informed Consent

The study design was in accordance with the guiding principles of the Declaration of Helsinki and was approved by the Institutional Ethics Committee of the University of Debrecen and the Ethics Committee of the National Medical Research Council. All patients or their relatives provided written informed consent.

### Blood Sampling and Laboratory Measurements

Peripheral blood samples were taken from all patients on admission, before the initiation of rt-PA infusion, and at 24 h post-lysis. In a subset of patients (*n* = 131), blood was also obtained immediately after administering full dose of rt-PA. Routine laboratory tests (ions, glucose level, renal and liver function tests, high-sensitivity C-reactive protein measurement, and complete blood count) were measured by standard laboratory methods (Roche Diagnostics, Mannheim, Germany, and Sysmex Europe GmbH, Hamburg, Germany). For studying specific hemostasis tests, blood samples were collected to vacutainer tubes containing 0.109 M sodium citrate (Becton Dickinson, Franklin Lane, NJ) and were processed immediately (centrifugation twice at 1,500 g, room temperature for 15 min). Screening tests of coagulation (prothrombin time, activated partial thromboplastin time, and thrombin time) and the determination of fibrinogen levels according to Clauss were performed on a BCS coagulometer using standard methods (Siemens Healthcare Diagnostic Products, Marburg, Germany). For the measurements of α2-PI activity and antigen levels, aliquots of citrated plasma were labeled with a unique code and stored at −80°C until analysis in batches. The buffy coat of blood samples was used to extract genomic DNA based on standard protocols (QIAamp DNA Blood Mini Kit, Qiagen, Hilden, Germany). The α2-PI Arg6Trp (rs2070863) polymorphism was identified by real-time PCR using fluorescence resonance energy transfer detection and melting curve analysis on a LightCycler® 480 instrument as described previously (Roche Diagnostics GmbH, Mannheim, Germany; primers are available from the authors upon request) (10). α2-PI activity and antigen level assays as well as the determination of α2-PI Arg6Trp genotype were carried out by investigators blinded to patient identification and clinical data.

### α2-Plasmin Inhibitor Activity and Antigen Assays

Functional α2-PI activity was measured from stored plasma samples using the Berichrom α2-PI activity chromogenic assay on a BCS coagulometer following the manufacturer's instructions (Siemens Healthcare Diagnostic Products, Marburg, Germany). The assay principle is the following: patient plasma containing α2-PI is incubated with the reagent that contains plasmin in excess. Plasmin becomes rapidly inactivated by α2-PI, and residual plasmin activity is measured by an amidolytic assay based on the cleavage of the plasmin-specific chromogenic substrate (D-norvalil-cyclohexylalanil-lysil-nitroanilid). α2-PI activity inversely correlates with the changes in absorption at 405 nm.

Total α2-PI antigen levels were measured by an in-house ELISA as previously described (39). This assay detects all forms of α2-PI and is not influenced by the presence of plasmin-antiplasmin complexes (reference range of plasma α2-PI antigen levels: 48–85 mg/L).

### Statistical Analysis

Statistical analysis was performed using the Statistical Package for Social Sciences (SPSS, Version 26.0, Chicago, IL) and GraphPad Prism 8.0 (GraphPad Prism Inc., La Jolla, CA). Shapiro–Wilk test was used to assess the normality of the data. Mann–Whitney U-test was performed for two-group analyses. In case of paired data, Wilcoxon signed-rank test was applied. Depending on the normality of data, ANOVA with Bonferroni *post-hoc* test or Kruskal–Wallis analysis with Dunn–Bonferroni *post-hoc* analysis was applied for multiple group comparisons. Spearman's correlation coefficient was used to determine the strength of correlation between continuous variables. Differences between categorical variables were assessed by χ^2^ test or by Fisher's exact where appropriate. The Kaplan–Meier method was applied to plot survival vs. non-survival of patients based on their α2-PI levels. Survival curves were compared using the log-rank test. Binary backward logistic regression models were used to determine independent predictors of mortality and long-term functional outcomes. Adjustments of the models were based on the results of univariate statistical analyses of baseline characteristics between groups (Mann–Whitney *U*-test, χ^2^ test, or Fisher's exact), previous literature, and methodological principles. The results of the logistic regression analysis were expressed as odds ratio (OR) and 95% confidence interval (CI). A *p* < 0.05 was considered statistically significant.

## Results

A total of 421 AIS patients receiving intravenous thrombolysis were included in the study. Baseline characteristics of patients and stroke outcomes are shown in Table 1. Median age of the cohort was 68 (IQR: 60–77) years, and 57.2% were men. Median NIHSS on admission was 7 (IQR: 4–11). Median time from symptom onset to treatment with rt-PA was 150 (IQR: 115–185) min. The most frequent cerebrovascular risk factor was hypertension (82.2 %). Favorable short- and long-term outcome was achieved in 45.2% and 48.6% of patients, respectively, excluding cases with post-lysis ICH. ICH occurred in 34 patients (8.1%), in 14 cases (3.3 % of total cohort) it was considered SICH, and in 20 patients (4.8% of total cohort) aSICH.

**Table 1:** Baseline characteristics and outcomes of enrolled patients.

	***n***
Number of patients	421
Age, y, median (IQR)	68 (60–77)
Male sex, n (%)	241 (57.2)
NIHSS on admission, median (IQR)	7 (4–11)
**Cerebrovascular risk factors, n (%)**	
Arterial hypertension	346 (82.2)
Atrial fibrillation	86 (20.4)
Diabetes mellitus	118 (28.0)
Hyperlipidaemia	267 (63.4)
Active smoker	128 (30.4)
Previous stroke, TIA	114 (27.1)
BMI, kg/m^2^, median (IQR)	27.4 (24.5–31.7)
**Medication at enrollment, n (%)**	
Antihypertensive therapy	209 (49.6)
Antiplatelet medication	173 (41.1)
Oral anticoagulant	22 (5.2)
Lipid lowering therapy	110 (26.1)
Antidiabetic therapy	69 (16.4)
**Laboratory measurements on admission, median (IQR)**	
WBC, G/L	7.9 (6.3–9.7)
Platelet count, G/L	217 (180–262)
Serum glucose, mmol/L	6.5 (5.6–8.1)
hsCRP, mg/L	2.9 (1.6–6.0)
INR	0.98 (0.93–1.02)
APTT, sec	27.8 (25.9–30.7)
α2-plasmin inhibitor activity, %	102 (93.0–111.0)
α2-plasmin inhibitor antigen, mg/L	59.6 (54.0–67.3)
**Stroke etiology (TOAST), n (%)**	
Large-artery atherosclerosis	146 (34.8)
Small-vessel occlusion	76 (18.0)
Cardioembolic	54 (12.8)
Other/undetermined	145 (34.4)
**Imaging data, n (%)**	
ASPECTS on admission	
0–7	6 (1.4)
8–10	268 (63.7)
ASPECTS at 24 h after thrombolysis	
0–7	50 (11.9)
8–10	225 (53.4)
**Thrombolysis (i.v. rt-PA) treatment, median (IQR)**	
Duration of thrombolysis, min	60 (60–60)
Time from symptom onset to treatment, min	150 (115–185)
rt-PA dose, mg	68 (58–81)
**Outcomes**, ***n*** **(%)**	
**Short-term outcome (Δ** **NIHSS, day 7)** ^*^	
Favorable (at least −4 points or day 7 NIHSS = 0)	175 (45.2)
Unchanged status (±3 points)	159 (41.0)
Unfavorable (at least +4 points or death)	46 (11.9)
Undetermined	7 (1.8)
**Long-term outcome (mRS, day 90)** ^*^	
Favorable (mRS 0–1)	188 (48.6)
Unfavorable (mRS 2–6)	157 (40.6)
Undetermined	42 (10.8)
**Intracerebral hemorrhage, ICH (ECASS II, 24 h)**	
No ICH	387 (91.9)
aSICH	20 (4.8)
SICH	14 (3.3)

*Data are medians (interquartile ranges) or n (%)*.

*APTT, activated partial thromboplastin time; aSICH, asymptomatic intracerebral hemorrhage; ASPECTS, Alberta Stroke Program Early CT Score; ECASS II, European Cooperative Acute Stroke Study-II; hsCRP, high sensitive C-reactive protein measurement; ICH, intracerebral hemorrhage; INR, international normalized ratio; IQR, interquartile ranges; mRS, modified Rankin Scale; NIHSS, National Institutes of Health Stroke Scale; SICH, symptomatic intracerebral hemorrhage; rt-PA, recombinant tissue plasminogen activator; TIA, transient ischemic attack; TOAST, Trial of ORG 10,172 in Acute Stroke Treatment; WBC, white blood cell count*.

* *Excluding patients with post-lysis intracerebral hemorrhage (n = 34)*.

### The Effect of Thrombolysis on α2-Plasmin Inhibitor Levels

**Figure 1:**
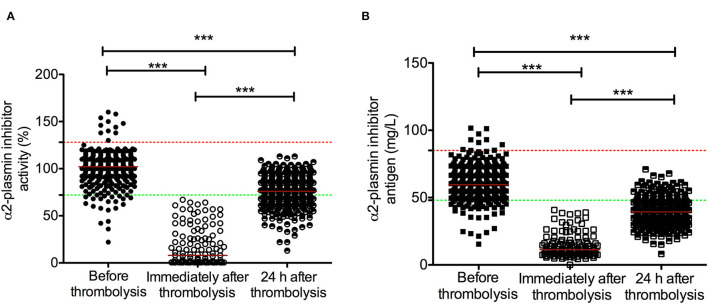
α2-plasmin inhibitor activity **(A)** and antigen levels **(B)** in acute ischemic stroke patients during the course of rt-PA thrombolysis. α2-plasmin inhibitor activity (circles) and antigen levels (squares) were assessed on admission (solid symbols), immediately after thrombolysis (immediately after administering full dose of rt-PA, empty symbols) and 24 h after thrombolysis (half-colored symbols). Red horizontal lines indicate median in each group. Upper and lower limits of reference are indicated with red and green dashed lines, respectively. rt-PA, recombinant tissue plasminogen activator. ^***^p < 0.001.

**Figure 2:**
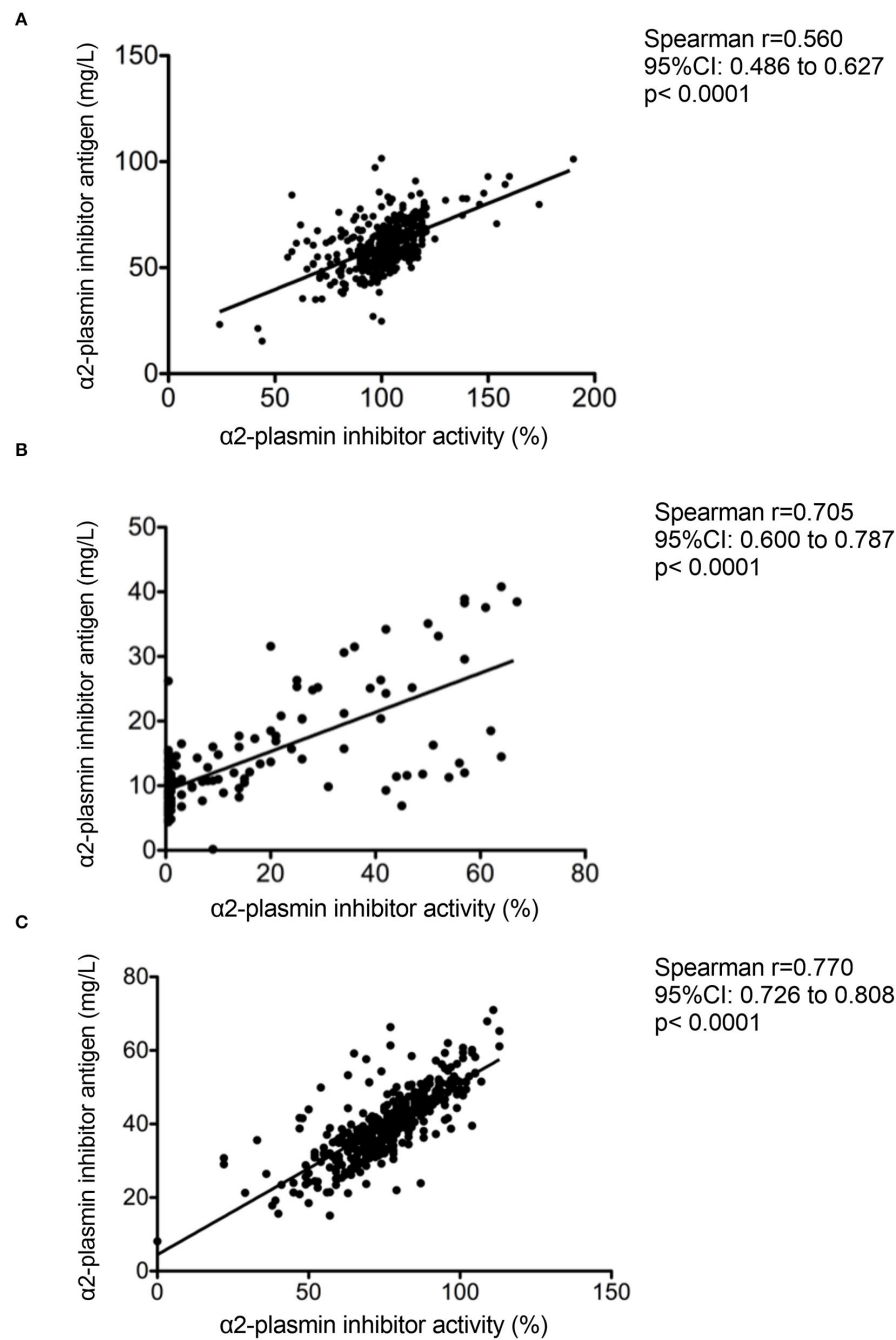
Correlation between α2-plasmin inhibitor activity and antigen levels as assessed on admission **(A)** immediately after thrombolysis **(B)** and 24 h after rt-PA thrombolysis **(C)** in acute ischemic stroke patients.

α2-PI activity and antigen levels before, immediately after, and 24 h after thrombolysis are shown on Figure 1. Admission α2-PI activity and antigen levels showed a surprisingly wide distribution, but the majority of patients had α2-PI levels within the reference range. Both α2-PI activity and antigen levels showed a highly significant decrease immediately after thrombolysis, indicating a rapid complex formation and consumption of the protein by the generated plasmin during the procedure. As shown in Figure 1, α2-PI activity and antigen levels of all patients were below the lower limit of reference when measured immediately after thrombolysis (α2-PI activity median: 8 [IQR: 1–29] %; α2-PI antigen median: 11.3 [IQR: 8.2–17.3] mg/L). Twenty-four hours after thrombolysis, α2-PI activity and antigen levels were substantially increased but were still below admission values (α2-PI activity median: 76 [66–86] %; α2-PI antigen median: 39.4 [IQR: 34.1–46.1] mg/L). Strongest correlation between α2-PI activity and antigen levels (r=0.770, 95%CI: 0.723–0.808, *p* < 0.001) was observed at 24 h after thrombolysis (Figure 2). Interestingly, the association between α2-PI activity and antigen levels on admission was found the be the weakest in this cohort (r=0.560, 95%CI: 0.486–0.627, *p* < 0.001). Among the baseline clinical and laboratory parameters (as listed in Table 1), both α2-PI activity and antigen levels showed a modest significant negative correlation with age (Spearman's r: −0.2244; 95%CI: −0.3168 to −0.1278, *p* < 0.001 and r: −0.3908; 95%CI: −0.4739 to −0.3008, *p* < 0.001, respectively). α2-PI activity, but not α2-PI antigen, showed a fair significant positive correlation with admission fibrinogen levels (r: 0.3623; 95%CI: 0.2723 to 0.4460, *p* < 0.001). α2-PI activity and antigen levels were significantly higher in active smokers and in women.

### Admission α2-Plasmin Inhibitor Levels and Stroke Characteristics

**Figure 3:**
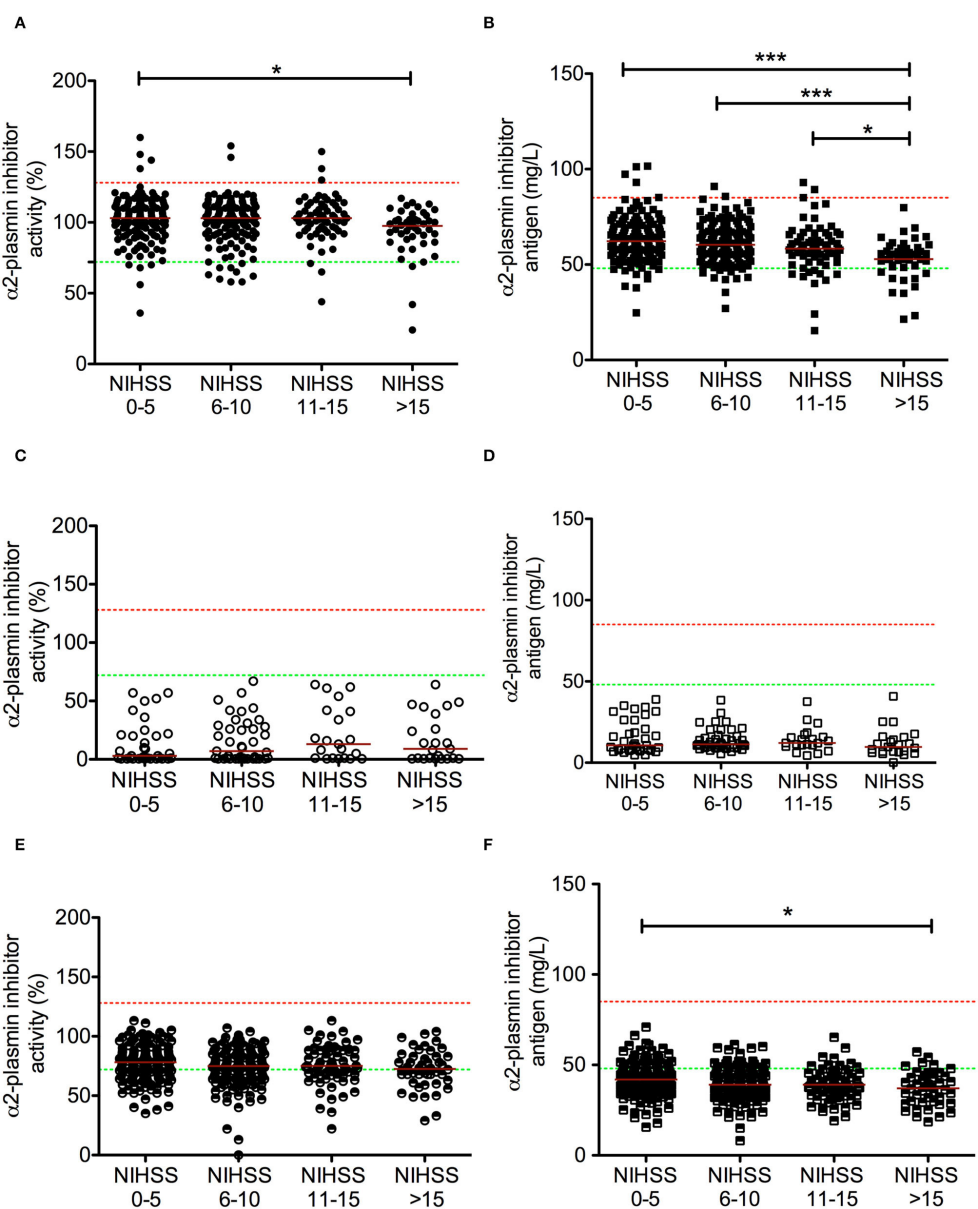
Association between α2-plasmin inhibitor activity and antigen levels at different time points during rt-PA thrombolysis with stroke severity on admission. α2-plasmin inhibitor activity was assessed on admission [**(A)** solid circles], immediately after thrombolysis [immediately after administering full dose of rt-PA, **(C)** empty circles], and 24 h after thrombolysis [**(E)** half-colored circles]. α2-plasmin inhibitor antigen levels were assessed on admission [**(B)** solid squares], immediately after thrombolysis [immediately after administering full dose of rt-PA, **(D)** empty squares], and 24 h after thrombolysis [**(F)** half-colored squares]. Red horizontal lines indicate median in each group. Stroke severity on admission was grouped according to NIHSS. Upper and lower limits of reference are indicated with red and green dashed lines, respectively. NIHSS, National Institutes of Health Stroke Scale, rt-PA, recombinant tissue plasminogen activator. *p < 0.05, ***p < 0.001.

α2-PI levels on admission showed a significant association with stroke severity (Figure 3). Those patients who suffered more severe stroke based on their NIHSS value on admission demonstrated significantly lower α2-PI levels (Figures 3A,B). The stepwise inverse association with stroke severity was found to be more profound for α2-PI antigen levels (Figure 3B), and a similar but weaker association was found again in samples obtained at 24 h post-lysis (Figure 3F). α2-PI levels after thrombolysis did not show a significant association with stroke severity (Figures 3C,D). Admission α2-PI antigen levels were the highest in case of small vessel infarcts and lowest in strokes of cardio-embolic origin (α2-PI antigen median: 61.8 [IQR: 56.3–72.8] mg/L vs. 56.6 [IQR: 52.3–64.2] mg/L, respectively, *p* = 0.024).

### α2-Plasmin Inhibitor Levels During Thrombolysis and Stroke Outcomes

**Figure 4:**
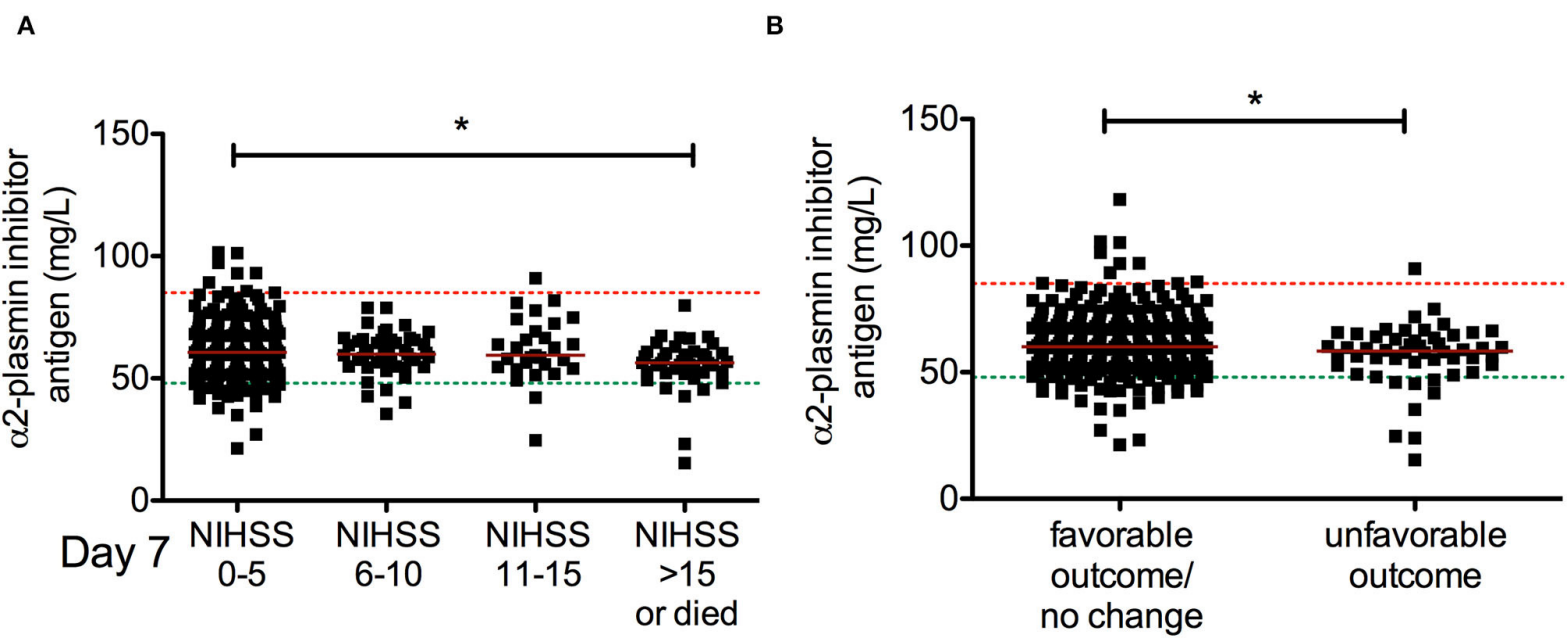
Association between α2-plasmin inhibitor antigen levels on admission and short-term outcome of thrombolysis. α2-plasmin inhibitor antigen levels were assessed on admission and compared to stroke severity on day 7 according to NIHSS **(A)** or to the change in the NIHSS by day 7 **(B)**. Favorable outcome (neurologic improvement) is defined as a decrease in NIHSS score by at least 4 points or to 0 by day 7, while an increase in NIHSS score by at least 4 points is defined as unfavorable outcome. Upper and lower limits of reference are indicated with red and green dashed lines, respectively. Red horizontal lines indicate median in each group. NIHSS, National Institutes of Health Stroke Scale, rt-PA, recombinant tissue plasminogen activator. *p < 0.05.

**Figure 5:**
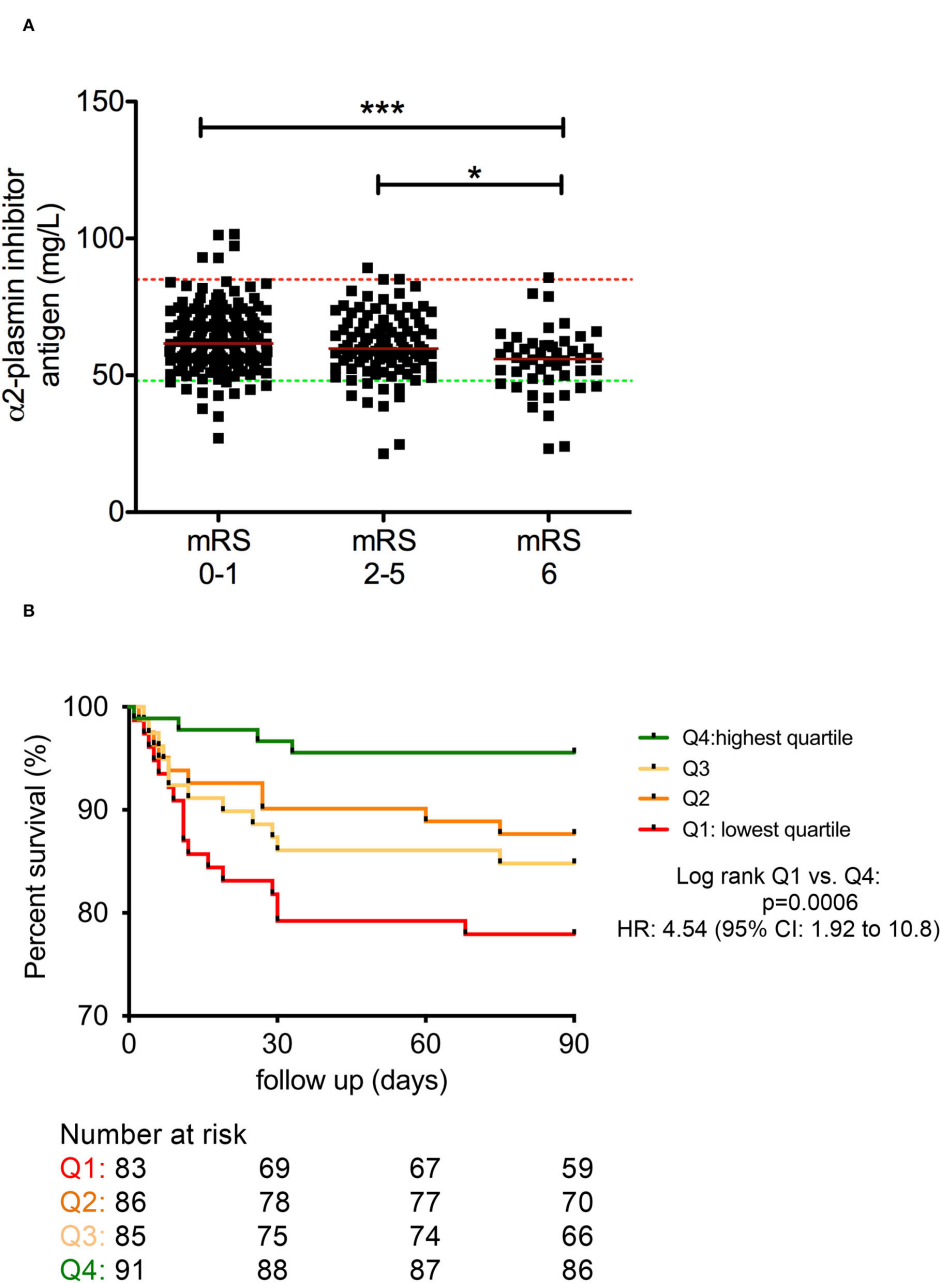
Association between α2-plasmin inhibitor antigen levels on admission and long-term outcome of thrombolysis **(A)**. Kaplan–Meier survival curves of patients based on quartiles of α2-plasmin inhibitor antigen levels as measured on admission **(B)**. α2-plasmin inhibitor antigen levels were assessed on admission (solid squares) and compared to long-term functional outcomes of thrombolysis based on the modified Rankin scale (mRS) **(A)**. Red horizontal lines indicate median in each group. Upper and lower limits of reference are indicated with red and green dashed lines, respectively. mRS 0–1 was considered as favorable outcome, and mRS 6 indicates mortality. Kaplan–Meier survival curves of patients are indicated according to quartiles of α2-plasmin inhibitor antigen levels on admission **(B)**. The number at risk is indicated below the graph. Upper quartile (Q4, green line), middle quartiles (Q3 and Q2, light orange and orange lines, respectively), and lowest quartile (Q1, red line) results were grouped based on the following cutoff values: 67.4 mg/L, 60 mg/L, and 54.1 mg/L α2-plasmin inhibitor antigen level, respectively. HR, hazard ratio; mRS, modified Rankin Scale. *p < 0.05, ***p < 0.001.

Admission α2-PI antigen levels showed a significant association with short-term outcomes of stroke (Figure 4). Significantly lower α2-PI antigen levels were found on admission in patients with more severe stroke at 7 days post-lysis (Figure 4A). In patients who demonstrated unfavorable outcome at 7 days post-lysis, α2-PI antigen levels on admission were significantly lower as compared to those improving or showing no change in their status (favorable outcome/no change group median: 60.4 [IQR: 54.5–68.8] mg/L vs. unfavorable outcome group median: 58.0 [IQR: 49.6–64.1] mg/L, p=0.045), although it must be noted that the difference between the median of the two groups was marginal (Figure 4B). α2-PI antigen levels after thrombolysis and α2-PI activity as measured at any time points were not associated with short-term outcomes. Similarly, long-term functional outcomes and mortality at 90 days were found to be associated only with α2-PI antigen levels on admission (Figure 5A). Of note, the extent of decrease between pre- and post-thrombolysis α2-PI activity or antigen levels did not show an association with outcomes (data not shown). Patients who died or had unfavorable long-term outcomes (mRS 2–5) had a significantly lower α2-PI antigen levels on admission as compared to patients with favorable long-term outcome at 90 days after the event (mRS 0–1 median: 61.6 [IQR: 55.9–70.5] mg/L vs. mRS 2–5 median: 59.7 [IQR: 54.5–69.1] mg/L vs. mRS 6 median: 56.0 [IQR: 48.5–61.0] mg/L, *p* < 0.001). In a Kaplan–Meier survival analysis, those patients who presented with α2-PI antigen level in the highest quartile on admission showed significantly better survival as compared to those with α2-PI antigen level in the lowest quartile on admission (HR: 4.54; 95%CI: 1.92–10.8, *p* < 0.001) (Figure 5B).

Binary backward logistic regression models (model 1: including hyperlipidemia, BMI, diabetes mellitus, sex, and CRP) revealed that α2-PI antigen level in the lowest quartile on admission is a significant predictor of unfavorable long-term outcomes (mRS 3–6) (OR: 2.10; 95%CI: 1.21–3.66, p=0.008) and death (mRS = 6) 3 months after thrombolysis (OR: 2.22, 95%CI: 1.15–4.31, *p* = 0.018) (Table 2). However, when age and NIHSS were also introduced in the models (Table 2, Models 2 and 3), the effect of α2-PI antigen level diminished and only age and NIHSS on admission remained as independent predictors of both outcomes.

**Table 2:** Independent predictors of unfavorable outcome (mRS 2–6) and mortality (mRS 6) at 90 days post-lysis.

	**Unfavorable outcome**			**Mortality at 90 days post-lysis**		
	**(mRS 0–1 vs. mRS 2–6)**			**(mRS 0–5 vs. mRS 6)**		
	**OR**	**95%CI**	***p***	**OR**	**95%CI**	***p***
**Model 1^§^**
α2-plasmin inhibitor antigen lowest quartile	2.10	1.21–3.66	0.008	2.22	1.15–4.31	0.018
Diabetes mellitus	2.18	1.29–3.68	0.004			
CRP (per 1 mg/L)				1.02	0.99–1.04	0.099
**Model 2^#^**
Diabetes mellitus	2.10	1.22–3.62	0.008			
Age (per 1 year)	1.05	1.03–1.07	<0.001	1.11	1.07–1.15	<0.001
CRP (per 1 mg/L)				1.03	1.00–1.05	0.028
**Model 3^†^**
Age (per 1 year)	1.04	1.02–1.06	<0.001	1.08	1.04–1.27	<0.001
NIHSS (per 1 point)	1.17	1.10–1.24	<0.001	1.17	1.09–1.25	<0.001
Diabetes mellitus	2.05	1.16–3.62	0.014			

*Last step of backward multiple regression analysis is provided*.

§ *Model 1 included sex, hyperlipidemia, BMI, diabetes mellitus, CRP, and α2-plasmin inhibitor antigen in the lowest quartile (threshold: <54.1 mg/L)*.

# *Model 2 included Model 1 and age*.

† *Model 3 included Model 2 and NIHSS*.

*95%CI, 95% confidence interval; CRP, C-reactive protein measurement; NIHSS, National Institutes of Health Stroke Scale; OR, odds ratio; mRS, modified Rankin Scale*.

**Figure 6:**
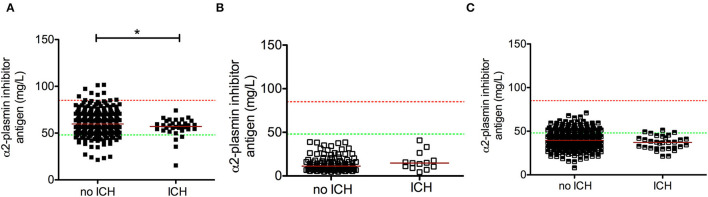
Association between α2-plasmin inhibitor antigen levels and post-lysis intracerebral bleeding (ICH). α2-plasmin inhibitor antigen levels were assessed on admission **(A)**, immediately after thrombolysis **(B)**, and 24 h after rt-PA thrombolysis **(C)**, and levels were compared in patients without or with intracerebral hemorrhage on day 1 post-lysis. Upper and lower limits of reference are indicated with red and green dashed lines, respectively. Red horizontal lines indicate median in each group. *p < 0.05.

In patients with therapy-related ICH (*n* = 32), admission α2-PI antigen levels were significantly lower as compared to those without hemorrhagic complications (Figure 6A). No such association was observed in the post-lysis samples of patients (Figures 6B,C) and in case of α2-PI activity at any measured time points (data not shown). However, difference between median α2-PI antigen levels on admission of those without or with post-lysis ICH was marginal (no ICH median: 59.8 [IQR: 54.0–68.0] mg/L vs. ICH: 57.0 [IQR: 53.4–61.7] mg/L, *p* = 0.036), and no correlation was observed between estimated post-lysis hematoma volumes and α2-PI antigen levels on admission (*r* = 0.207; 95%CI: −0.176 to 0.536, *p* = 0.272). α2-PI antigen or activity levels did not differ in subgroups of SICH or aSICH at any time points investigated (data not shown). As expected, NIHSS was significantly higher in patients with ICH as compared to those without post-lysis hemorrhage (median: 12 [IQR: 7–32] vs. 6.5 [IQR: 4–36], respectively, *p* < 0.001).

### α2-Plasmin Inhibitor Arg6Trp Polymorphism, Stroke Characteristics, and Outcomes

Genotype frequencies of α2-PI Arg6Trp polymorphism were consistent with Hardy–Weinberg equilibrium in the cohort (C = 0.8046; T = 0.1953) and were practically identical to allele frequencies reported in the 1000 Genomes project for the European subgroup (C = 0.8012; T = 0.1988) (40). α2-PI Arg6Trp polymorphism had no effect on α2-PI activity or antigen levels at any measured time points and showed no association with stroke severity, etiology, or outcomes (data not shown).

## Discussion

In this prospective, observational study of 421 AIS patients undergoing thrombolysis, low α2-PI levels on admission were found to be associated with more severe strokes and significantly worse long-term outcomes and mortality. Here, we report that α2PI activity and antigen levels show a significant drop immediately post-lysis and increase to subnormal levels at 24 h post-event; however, post-lysis α2-PI levels showed no association with outcomes. Despite the fact that in a multivariate analysis, admission α2-PI levels did not prove to be an independent predictor of thrombolysis outcome, our results suggest that α2-PI incorporation into thrombi could be an important mechanism for the limited efficacy of rt-PA thrombolysis.

Understanding the reasons of thrombolysis failure is critical for improving acute stroke care. Although α2-PI is among the key regulators of fibrinolysis, little is known about its role in AIS thrombolysis outcome as yet. In the literature, only a few studies have been published on the potential association between α2-PI levels and AIS thrombolysis outcomes, with controversial results (12, 25, 26, 41, 42). In a study including 63 AIS patients, baseline levels of α2-PI activity correlated well with the rate of recanalization (42). Patients who recanalized had lower α2-PI activity levels, and α2-PI level was described to be the only predictive variable of recanalization, although it did not show an association with long-term outcomes. On the contrary, in studies by others, although a decrease in α2-PI levels was found after thrombolysis, α2-PI levels showed no association with therapy outcomes (25, 26, 41). As it was pointed out by a recent systematic review, controversial data on markers of hemostasis and fibrinolysis and the outcome in AIS thrombolysis may arise from numerous methodological issues (15). First, most studies include relatively few patients lacking statistical power and only a handful of papers are published in the literature where at least 100 patients are included. Another critical factor is the time interval between stroke onset and blood sampling, as in the majority of studies a baseline blood sample is collected within 24 h after stroke onset, that is, a fairly wide time interval. Moreover, in patients receiving thrombolysis, therapy is provided during this period; thus, it is essential to differentiate between results gained from pre- or post-thrombolysis blood samples. Ideally, a hemostasis biomarker should be assessed before the initiation of thrombolysis treatment in AIS patients, despite the fact that sample collection might be a challenging task due to the short time window of treatment. Finally, depending on the research question, more than one sampling time point could be necessary to draw sound conclusions. In this prospective observational study, we were able to enroll 421 AIS patients undergoing thrombolysis, which makes this cohort one of the largest published as yet. Blood samples were collected from all patients before and 24 h after thrombolysis, while in a subset of patients we also aimed at collecting samples immediately post-lysis. α2-PI levels were tested at all time points by a functional test and an antigen assay detecting all four isoforms. Due to the natural heterogeneity of this protein, data derived from both assays could shed light on the action of α2-PI during AIS thrombus formation and provide information on the inhibitory effect of α2-PI on the excess plasmin generated during rt-PA therapy. Similarly to previous reports, α2-PI activity and antigen results showed good correlation in this cohort (43). Interestingly, on admission, only moderately strong correlation was found between the activity and antigen assay results, which then improved post-lysis. This might arise from the fact that the incorporation of α2-PI into the thrombus by FXIIIa primarily involves PB-α2-PI (10, 43). It has been suggested by previous reports that the commercially available chromogenic α2-PI activity assay used in this study is mainly sensitive to the levels of PB-α2-PI, the more active and kinetically faster plasmin inhibitor (44, 45). On the contrary, the α2-PI antigen test used captures all forms of α2-PI to an equal extent; therefore, it is surmised to fully demonstrate the extent of α2-PI consumption. This might be the reason for the stronger association between α2-PI antigen levels and stroke severity observed in this cohort as compared to α2-PI activity and might explain the link between admission α2-PI antigen levels and outcomes. Further studies are required to understand the extent of incorporation of various α2-PI isoforms into thrombi and their relation to thrombus burden and treatment outcomes in AIS patients.

Based on our results, there is a considerable consumption of α2-PI in patients with more severe strokes, as a significant stepwise negative association was found between α2-PI antigen levels and stroke severity. Significantly lower admission α2-PI antigen levels were found in patients with unfavorable short- and long-term outcomes; moreover, admission α2-PI antigen levels in the lowest quartile showed a significant association with the long-term mortality of patients. However, in a multivariate analysis including all relevant factors determining long-term outcomes, the significant effect of α2-PI was diminished and only age and NIHSS remained in the model as most important predictors of long-term outcomes. These results suggest that although the incorporation of α2-PI into intracerebral thrombi and thus the consumption of the protein is associated with stroke severity and most probably with thrombus burden as well, apparently, α2-PI cannot be considered an independent biomarker of therapy outcome. Nevertheless, these results suggest that α2-PI could play a role in the pathophysiology of thrombolysis failure and may be an important factor contributing to the inefficacy of therapy in case of severe strokes. Consistent with this finding, we have shown in previous work that the extent of α2-PI incorporation into fibrin clots was the lowest in AIS patients with favorable outcomes after thrombolysis (10). These results are in line with another recent work from our group where we showed that together with age and NIHSS, thrombus burden is the most important indicator of thrombolysis outcome, and the levels of a number of key hemostasis parameters measured on admission or 24 h later showed no association with treatment outcomes in a multivariate model (46).

Although it is biologically plausible that low α2-PI levels post-lysis could be responsible for therapy-associated ICH events, such association was not found in this cohort. α2-PI levels immediately post-lysis were below the lower limit of reference in all individuals tested in this cohort, yet bleeding complications were not directly related to levels of α2-PI at this time point. These results are in line with experimental studies suggesting that targeting α2-PI may be a novel paradigm for the dissolution of thrombi without compromising safety (47). Admission α2-PI levels were indeed significantly lower in patients with post-lysis ICH; however, difference between groups was very modest. As patients with post-lysis ICH presented with more severe stroke on admission, it can be surmised that the significant difference observed between the α2-PI levels of the two groups is related to differences in stroke severity. As α2-PI levels in the groups with or without ICH showed a considerable overlap on admission, moreover, α2-PI activity levels did not differ among groups, and it is unlikely that low α2-PI would be the only responsible factor for post-lysis ICH. α2-PI had no effect on the enlargement of the hematoma as α2-PI levels did not show a significant correlation with the estimated post-lysis hematoma volumes. On the contrary, it has been known from the literature that more severe strokes are associated with not only lysis failure, but also with a higher chance of bleeding complications, although the reasons for this association have not been clarified (48). We have shown in a previous report that based on the *in vitro* incorporation of α2-PI into fibrin clots, higher stroke severity was found to be associated with lower susceptibility to thrombolysis, together with a potentially higher risk of bleeding due to increased consumption of α2-PI. The balance of fibrinolysis in these cases might be shifted toward bleeding near the thrombus site, which, together with other factors, could result in less protection against the rt-PA induced hemostatic challenge.

Among the parameters influencing α2-PI heterogeneity, α2-PI Arg6Trp was tested in the cohort. Given the relatively large sample size, the effect of α2-PI Arg6Trp polymorphism on α2-PI levels, stroke characteristics, and treatment outcomes was assessed, but no associations were found. Only a few studies have investigated the relation between α2-PI Arg6Trp polymorphism and the risk or outcomes of arterial thrombotic events. Limited data based on these reports suggest that the polymorphism has no effect on the risk or outcomes of ischemic events. As we have shown most recently, besides α2-PI Arg6Trp polymorphism, an important modifying factor of the extent of α2-PI N-terminal cleavage is circulating APCE (sFAP) level (43). It can be surmised that the effect of α2-PI Arg6Trp should be considered in light of APCE levels; however, this measurement was out of the scope of our study.

## Limitations

The results of this study should be interpreted in the context of its limitations and strengths. Although a more complex picture on the pathophysiology of α2-PI during thrombolysis may have been gained by the measurement of α2-PI isoforms, this was out of the scope of this study. Due to the single-centered study design, the study had the advantage of uniform patient and sample handling; moreover, the proportion of patients lost to follow-up (1.8 and 10.8% for short-term and long-term outcome, respectively) was relatively low as compared to other studies of similar study design (15). On the contrary, patients receiving mechanical thrombectomy were not included in the study and therefore the results cannot be directly translated to current treatment practices of AIS patients with LVO.

## Conclusions

In this cohort of AIS patients, levels of α2-PI changed dramatically during thrombolysis, but post-lysis α2-PI levels showed no association with treatment outcomes or safety. Significantly lower α2-PI levels were found on admission in AIS patients presenting with more severe stroke. Significantly lower admission α2-PI antigen levels were found in patients with unfavorable short- and long-term outcomes; however, in a multivariate analysis including all relevant factors determining long-term outcomes, the significant effect of α2-PI was diminished and only age and NIHSS remained in the model as most important predictors of long-term outcomes. Taken together, these data suggest that although α2-PI is a dominant inhibitor of physiologic fibrinolysis, its levels on admission are strongly related to stroke severity and are affected by age to some extent, and they are not independent predictors of outcomes. It can be surmised, however, that in case of more severe strokes, the extent of α2-PI incorporation into thrombi is higher, which could be an important mechanism for the limited efficacy of rt-PA thrombolysis in these cases.

## Data Availability Statement

The original contributions presented in the study are included in the article/supplementary materials, further inquiries can be directed to the corresponding author.

## Ethics Statement

The studies involving human participants were reviewed and approved by the Institutional Ethics Committee of the University of Debrecen and the Ethics Committee of the National Medical Research Council, Hungary. The patients/participants provided their written informed consent to participate in this study.

## Author Contributions

ES and RO-K collected clinical samples, performed experiments, and analyzed and interpreted data. IS, KC-K, NV, IF, KF, and EB collected clinical data and analyzed and interpreted data. BB and LL performed preanalytical sample care and performed experiments. ÉK, LO, and LC analyzed and interpreted the data. ZB designed the research, analyzed and interpreted the data, and wrote the manuscript. All authors have read and approved the final manuscript.

## Funding

This study was supported by grants from the National Research, Development and Innovation Fund (K120042, FK128582, and K120633), by GINOP-2.3.2-15-2016-00043, the Eötvös Loránd Research Network (ELKH-DE Cerebrovascular and Neurodegenerative Research Group), and the New National Excellence Program (UNKP-21-4-1).

## Conflict of Interest

The authors declare that the research was conducted in the absence of any commercial or financial relationships that could be construed as a potential conflict of interest.

## Publisher's Note

All claims expressed in this article are solely those of the authors and do not necessarily represent those of their affiliated organizations, or those of the publisher, the editors and the reviewers. Any product that may be evaluated in this article, or claim that may be made by its manufacturer, is not guaranteed or endorsed by the publisher.

## References

[1] Katan M Luft A. Global burden of stroke. Semin Neurol. (2018) 38:208–11. 10.1055/s-0038-1649503 29791947

[2] Powers WJ Rabinstein AA Ackerson T Adeoye OM Bambakidis NC Becker K. 2018 guidelines for the early management of patients with acute ischemic stroke: a guideline for healthcare professionals from the American Heart Association/American Stroke Association. Stroke. (2018) 49:e46–e110. 10.1161/STR.0000000000000158 29367334

[3] National National Institute of Neurological Disorders Stroke rt-PA SSG. Tissue plasminogen activator for acute ischemic stroke. N Engl J Med. (1995) 333:1581–7. 10.1056/NEJM199512143332401 7477192

[4] Hacke W Kaste M Bluhmki E Brozman M Davalos A Guidetti D. Thrombolysis with alteplase 3 to 4.5 hours after acute ischemic stroke. N Engl J Med. (2008) 359:1317–29. 10.1056/NEJMoa0804656 18815396

[5] Henderson SJ Weitz JI Kim PY. Fibrinolysis: strategies to enhance the treatment of acute ischemic stroke. J Thromb Haemost. (2018) 16:1932–40. 10.1111/jth.14215 29953716

[6] Nogueira RG Jadhav AP Haussen DC Bonafe A Budzik RF Bhuva P. Thrombectomy 6 to 24 hours after stroke with a mismatch between deficit and infarct. N Engl J Med. (2018) 378:11–21. 10.1056/NEJMoa1706442 29129157

[7] del Zoppo GJ Poeck K Pessin MS Wolpert SM Furlan AJ Ferbert A. Recombinant tissue plasminogen activator in acute thrombotic and embolic stroke. Ann Neurol. (1992) 32:78–86. 10.1002/ana.410320113 1642475

[8] Wahlgren N Ahmed N Davalos A Ford GA Grond M Hacke W. Thrombolysis with alteplase for acute ischaemic stroke in the Safe Implementation of Thrombolysis in Stroke-Monitoring Study (SITS-MOST): an observational study. Lancet. (2007) 369:275–82. 10.1016/S0140-6736(07)60149-4 17258667

[9] Lees KR Emberson J Blackwell L Bluhmki E Davis SM Donnan GA. Effects of alteplase for acute stroke on the distribution of functional outcomes: a pooled analysis of 9 trials. Stroke. (2016) 47:2373–9. 10.1093/med/9780199687039.003.0067_update_001 27507856 PMC5024752

[10] Bagoly Z Barath B Orban-Kalmandi R Szegedi I Bogati R Sarkady F. Incorporation of alpha2-plasmin inhibitor into fibrin clots and its association with the clinical outcome of acute ischemic stroke patients. Biomolecules. (2021) 11. 10.3390/biom11030347 33669007 PMC7996613

[11] Szekely EG Czuriga-Kovacs KR Bereczky Z Katona E Mezei ZA Nagy A. Low factor XIII levels after intravenous thrombolysis predict short-term mortality in ischemic stroke patients. Sci Rep. (2018) 8:7662. 10.1038/s41598-018-26025-z 29769590 PMC5955963

[12] Bagoly Z Szegedi I Kalmandi R Toth NK Csiba L. Markers of coagulation and fibrinolysis predicting the outcome of acute ischemic stroke thrombolysis treatment: a review of the literature. Front Neurol. (2019) 10:513. 10.3389/fneur.2019.00513 31316444 PMC6611415

[13] Orban-Kalmandi R Szegedi I Sarkady F Fekete I Fekete K Vasas N. A modified *in vitro* clot lysis assay predicts outcomes and safety in acute ischemic stroke patients undergoing intravenous thrombolysis. Sci Rep. (2021) 11:12713. 10.1038/s41598-021-92041-1 34135389 PMC8208992

[14] Szegedi I Nagy A Szekely EG Czuriga-Kovacs KR Sarkady F Lanczi LI. PAI-1 5G/5G genotype is an independent risk of intracranial hemorrhage in post-lysis stroke patients. Ann Clin Transl Neurol. (2019) 6:2240–50. 10.1002/acn3.50923 31637872 PMC6856768

[15] Donkel SJ Benaddi B Dippel DWJ Ten Cate H de Maat MPM. Prognostic hemostasis biomarkers in acute ischemic stroke. Arterioscler Thromb Vasc Biol. (2019) 39:360–72. 10.1161/ATVBAHA.118.312102 30700129 PMC6392207

[16] Bagoly Z Ariens RAS Rijken DC Pieters M Wolberg AS. Clot structure and fibrinolysis in thrombosis and hemostasis. Biomed Res Int. (2017) 2017:4645137. 10.1155/2017/4645137 29270431 PMC5705862

[17] Muszbek L Bagoly Z Bereczky Z Katona E. The involvement of blood coagulation factor XIII in fibrinolysis and thrombosis. Cardiovasc Hematol Agents Med Chem. (2008) 6:190–205. 10.2174/187152508784871990 18673233

[18] Chapin JC Hajjar KA. Fibrinolysis and the control of blood coagulation. Blood Rev. (2015) 29:17–24. 10.1016/j.blre.2014.09.003 25294122 PMC4314363

[19] Wiman B Collen D. On the mechanism of the reaction between human alpha 2-antiplasmin and plasmin. J Biol Chem. (1979) 254:9291–7. 10.1016/S0021-9258(19)86843-6 158022

[20] Abdul S Leebeek FW Rijken DC Uitte de Willige S.. Natural heterogeneity of alpha2-antiplasmin: functional and clinical consequences. Blood. (2016) 127:538–45. 10.1182/blood-2015-09-670117 26626994

[21] Muszbek L Bereczky Z Bagoly Z Komaromi I Katona E. Factor XIII: a coagulation factor with multiple plasmatic and cellular functions. Physiol Rev. (2011) 91:931–72. 10.1152/physrev.00016.2010 21742792

[22] Sakata Y Aoki N. Cross-linking of alpha 2-plasmin inhibitor to fibrin by fibrin-stabilizing factor. J Clin Invest. (1980) 65:290–7. 10.1172/JCI109671 6444305 PMC371366

[23] Fassbender K Dempfle CE Mielke O Schwartz A Daffertshofer M Eschenfelder C. Changes in coagulation and fibrinolysis markers in acute ischemic stroke treated with recombinant tissue plasminogen activator. Stroke. (1999) 30:2101–4. 10.1161/01.STR.30.10.2101 10512913

[24] Echouffo-Tcheugui JB Woodward M Kengne AP. Predicting a post-thrombolysis intracerebral hemorrhage: a systematic review. J Thromb Haemost. (2013) 11:862–71. 10.1111/jth.12186 23469771

[25] Sun X Berthiller J Derex L Trouillas P Diallo L Hanss M. Post-thrombolysis haemostasis changes after rt-PA treatment in acute cerebral infarct. Correlations with cardioembolic aetiology and outcome. J Neurol Sci. (2015) 349:77–83. 10.1016/j.jns.2014.12.029 25619569

[26] Marti-Fabregas J Borrell M Cocho D Martinez-Ramirez S Martinez-Corral M Fontcuberta J. Change in hemostatic markers after recombinant tissue-type plasminogen activator is not associated with the chance of recanalization. Stroke. (2008) 39:234–6. 10.1161/STROKEAHA.107.493767 18048863

[27] Lee KN Jackson KW Christiansen VJ Chung KH McKee PA A. novel plasma proteinase potentiates alpha2-antiplasmin inhibition of fibrin digestion. Blood. (2004) 103:3783–8. 10.1182/blood-2003-12-4240 14751930

[28] Bangert K Johnsen AH Christensen U Thorsen S. Different N-terminal forms of alpha 2-plasmin inhibitor in human plasma. Biochem J. (1993) 291:623–5. 10.1042/bj2910623 8484741 PMC1132569

[29] Sumi Y Ichikawa Y Nakamura Y Miura O Aoki N. Expression and characterization of pro alpha 2-plasmin inhibitor. J Biochem. (1989) 106:703–7. 10.1093/oxfordjournals.jbchem.a122920 2606916

[30] Christiansen VJ Jackson KW Lee KN McKee PA. The effect of a single nucleotide polymorphism on human alpha 2-antiplasmin activity. Blood. (2007) 109:5286–92. 10.1182/blood-2007-01-065185 17317851 PMC1890835

[31] Wiman B. Affinity-chromatographic purification of human alpha 2-antiplasmin. Biochem J. (1980) 191:229–32. 10.1042/bj1910229 6907016 PMC1162201

[32] Pechlivani N Kearney KJ Ajjan RA. Fibrinogen and antifibrinolytic proteins: interactions and future therapeutics. Int J Mol Sci. (2021) 22. 10.3390/ijms222212537 34830419 PMC8625824

[33] European Stroke Organisation Executive Committee E. S. O. Writing Committee. Guidelines for management of ischaemic stroke and transient ischaemic attack (2008). Cerebrovasc Dis. (2008) 25:457–507. 10.1159/000131083 18477843

[34] Aviv RI Mandelcorn J Chakraborty S Gladstone D Malham S Tomlinson G. Alberta Stroke Program Early CT Scoring of CT perfusion in early stroke visualization and assessment. AJNR Am J Neuroradiol. (2007) 28:1975–80. 10.3174/ajnr.A0689 17921237 PMC8134254

[35] Brott T Adams HP Jr Olinger CP Marler JR Barsan WG Biller J. Measurements of acute cerebral infarction: a clinical examination scale. Stroke. (1989) 20:864–70. 10.1161/01.STR.20.7.864 2749846

[36] Adams HP Jr Bendixen BH Kappelle LJ Biller J Love BB Gordon DL. Classification of subtype of acute ischemic stroke. Definitions for use in a multicenter clinical trial. TOAST. Trial of Org 10172 in Acute Stroke Treatment. Stroke. (1993) 24:35–41. 10.1161/01.STR.24.1.35 7678184

[37] Simonsen CZ Schmitz ML Madsen MH Mikkelsen IK Chandra RV Leslie-Mazwi T. Early neurological deterioration after thrombolysis: clinical and imaging predictors. Int J Stroke. (2016) 11:776–82. 10.1177/1747493016650454 27188241

[38] Hacke W Kaste M Fieschi C Toni D Lesaffre E von Kummer R. Intravenous thrombolysis with recombinant tissue plasminogen activator for acute hemispheric stroke. The European Cooperative Acute Stroke Study (ECASS). JAMA. (1995) 274:1017–25. 10.1001/jama.1995.03530130023023 7563451

[39] Teraz-Orosz A Csapo A Bagoly Z Szekely EG Toth E Kovacs B. A new ELISA method for the measurement of total alpha2-plasmin inhibitor level in human body fluids. J Immunol Methods. (2019) 471:27–33. 10.1016/j.jim.2019.05.004 31129263

[40] 1000 Genomes Project Consortium; Auton A Brooks LD Durbin RM Garrison EP Kang HM. A global reference for human genetic variation. Nature. (2015) 526:68–74. 26432245 10.1038/nature15393 PMC4750478

[41] Cocho D Borrell M Marti-Fabregas J Montaner J Castellanos M Bravo Y. Pretreatment hemostatic markers of symptomatic intracerebral hemorrhage in patients treated with tissue plasminogen activator. Stroke. (2006) 37:996–9. 10.1161/01.STR.0000206461.71624.50 16497981

[42] Marti-Fabregas J Borrell M Cocho D Belvis R Castellanos M Montaner J. Hemostatic markers of recanalization in patients with ischemic stroke treated with rt-PA. Neurology. (2005) 65:366–70. 10.1212/01.wnl.0000171704.50395.ba 16087899

[43] Barath B Bogati R Miklos T Kallai J Mezei ZA Bereczky Z. Effect of alpha2-plasmin inhibitor heterogeneity on the risk of venous thromboembolism. Thromb Res. (2021) 203:110–6. 10.1016/j.thromres.2021.05.003 33992873

[44] Uitte de Willige S Miedzak M Carter AM Lisman T Rosendaal FR Grant PJ. Proteolytic and genetic variation of the alpha-2-antiplasmin C-terminus in myocardial infarction. Blood. (2011) 117:6694–701. 10.1182/blood-2010-11-320325 21505192

[45] Clemmensen I Thorsen S Mullertz S Petersen LC. Properties of three different molecular forms of the alpha 2 plasmin inhibitor. Eur J Biochem. (1981) 120:105–12. 10.1111/j.1432-1033.1981.tb05675.x 6458494

[46] Szegedi I Orban-Kalmandi R Nagy A Sarkady F Vasas N Sik M. Decreased clot burden is associated with factor XIII Val34Leu polymorphism and better functional outcomes in acute ischemic stroke patients treated with intravenous thrombolysis. PLoS ONE. (2021) 16:e0254253. 10.1371/journal.pone.0254253 34234378 PMC8263307

[47] Singh S Saleem S Reed GL. Alpha2-antiplasmin: the devil you don't know in cerebrovascular and cardiovascular disease. Front Cardiovasc Med. (2020) 7:608899. 10.3389/fcvm.2020.608899 33426005 PMC7785519

[48] Jensen M Schlemm E Cheng B Lettow I Quandt F Boutitie F. Clinical characteristics and outcome of patients with hemorrhagic transformation after intravenous thrombolysis in the WAKE-UP trial. Front Neurol. (2020) 11:957. 10.3389/fneur.2020.00957 32982951 PMC7483750

